# Point shear wave elastography for non-invasive detection of advanced fibrosis in metabolic dysfunction-associated steatotic liver disease: a prospective histologically validated study

**DOI:** 10.1186/s12876-026-05123-7

**Published:** 2026-07-15

**Authors:** Wolfgang Kratzer, Andreas Binzberger, Stefan G. Kauschke, Finn Dolze, Markus Werner, Thomas F. E. Barth, Heike Neubauer, Eric Simon, Andreas David Brunner, Benedikt Haggenmüller, Mark M. Haenle

**Affiliations:** 1 Abteilung für Innere Medizin I, Universitätsklinikum Ulm, Albert-Einstein-Allee 23, Ulm, 89081 Germany; 2 Boehringer Ingelheim Pharma GmbH & Co. KG, Biberach, Germany; 3 Institut für Pathologie, Universität Ulm, Ulm, Germany; 4 Institute of Diagnostic and Interventional Radiology, Universitätsklinikum Ulm, Ulm, Germany

**Keywords:** MASLD, Liver fibrosis, Point shear wave elastography, ARFI, Ultrasound, Non-invasive diagnosis

## Abstract

**Background:**

Metabolic dysfunction–associated steatotic liver disease (MASLD) is the most common chronic liver disease worldwide, and fibrosis stage is the strongest predictor of liver-related outcomes. Point shear wave elastography (pSWE), based on acoustic radiation force impulse technology, is widely available in routine ultrasound systems; however, histologically validated data in well-characterized MASLD cohorts remain limited. This study aimed to evaluate the diagnostic performance of pSWE for the detection of advanced fibrosis (≥ F3) using histology as the reference standard.

**Methods:**

In this prospective single-center study, adult patients with MASLD undergoing clinically indicated liver biopsy were included. Liver stiffness was assessed using pSWE on a Siemens ultrasound system. Histological fibrosis staging (F0–F4) was performed by three blinded liver pathologists. Diagnostic performance for advanced fibrosis (≥ F3) was evaluated using receiver operating characteristic (ROC) analysis. Clinically applicable rule-in and rule-out thresholds were determined using predefined sensitivity- and specificity-based criteria.

**Results:**

A total of 59 patients with MASLD and numerically evaluable pSWE measurements were included. Advanced fibrosis (≥ F3) was present in 18/59 patients (30.5%). pSWE demonstrated excellent diagnostic performance for the detection of advanced fibrosis, with an AUROC of 0.90 (95% CI 0.82–0.97). A threshold of 1.255 m/s achieved 100% sensitivity and 100% negative predictive value, supporting a rule-out strategy. A threshold of 1.96 m/s achieved 97.6% specificity and 91.7% positive predictive value, supporting a rule-in strategy. Shear wave velocity correlated significantly with histological fibrosis stage (Spearman ρ = 0.68, *p* < 0.001).

**Conclusion:**

Point shear wave elastography provides reliable non-invasive detection of advanced fibrosis in MASLD. Technique-specific thresholds enable clinically meaningful rule-in and rule-out strategies and may support integration of pSWE into stepwise non-invasive fibrosis risk stratification pathways.

## Highlights

pSWE shows excellent diagnostic accuracy for advanced fibrosis in MASLD (AUROC 0.90).

Clinically applicable pSWE rule-in and rule-out thresholds were identified.

A threshold of 1.255 m/s reliably excludes advanced fibrosis (NPV 100%).

A threshold of 1.96 m/s identifies advanced fibrosis with high specificity (97.6%).

Prospective design with histological validation by three independent pathologists.

## Background

Metabolic dysfunction-associated steatotic liver disease (MASLD) represents the most common chronic liver disease worldwide and constitutes a rapidly increasing global health burden [1, 2]. Recent data from the Global Burden of Disease Study 2023 demonstrate a substantial increase in MASLD prevalence over the past decades, with further marked growth projected until 2050 [2]. The disease spectrum ranges from simple steatosis to advanced fibrosis and cirrhosis. Fibrosis stage has consistently been identified as the strongest predictor of liver-related morbidity and mortality [3]. Current international guidelines recommend a stepwise, non-invasive approach for fibrosis risk stratification in MASLD [4, 5]. Simple serum-based scores such as the Fibrosis-4 Index (FIB-4) are widely used as first-line screening tools [6]. However, their diagnostic performance may be influenced by age and metabolic comorbidities, potentially leading to reduced accuracy in specific patient populations [7]. As a result, imaging-based assessment of liver stiffness has become an essential component of clinical decision-making in MASLD [8].

Ultrasound-based elastography techniques have emerged as widely used non-invasive tools for fibrosis assessment [9, 10]. Among these, point shear wave elastography (pSWE), based on acoustic radiation force impulse technology, is integrated into conventional ultrasound systems and is therefore broadly available in routine clinical practice [9, 10]. This widespread availability makes pSWE particularly attractive for implementation in real-world diagnostic algorithms. However, an important limitation of elastography-based methods is that liver stiffness values are not interchangeable across different techniques and ultrasound systems because of variations in measurement principles and signal processing [9, 10]. Consequently, technique-specific validation and threshold definition are essential before clinical implementation. While pSWE has increasingly been applied in clinical practice, histologically validated data in well-characterized MASLD cohorts remain limited, particularly regarding clinically applicable rule-in and rule-out thresholds. Recent studies have supported the clinical utility of pSWE in MASLD populations, including obese patients, but further histology-based validation in routine clinical settings is needed [11, 12]. Therefore, the aim of this study was to evaluate the diagnostic performance of pSWE for the detection of advanced fibrosis (≥ F3) in MASLD using histology as the reference standard and to establish clinically applicable thresholds for non-invasive risk stratification.

## Methods

### Study design and population

This prospective single-center study was conducted at a university-based interdisciplinary ultrasound center. Patient recruitment was performed between March 2020 and November 2025. Consecutive adult patients (≥ 18 years) with clinical suspicion of metabolic dysfunction-associated steatotic liver disease (MASLD) who underwent clinically indicated liver biopsy and ultrasound elastography were.

**Fig. 1:**
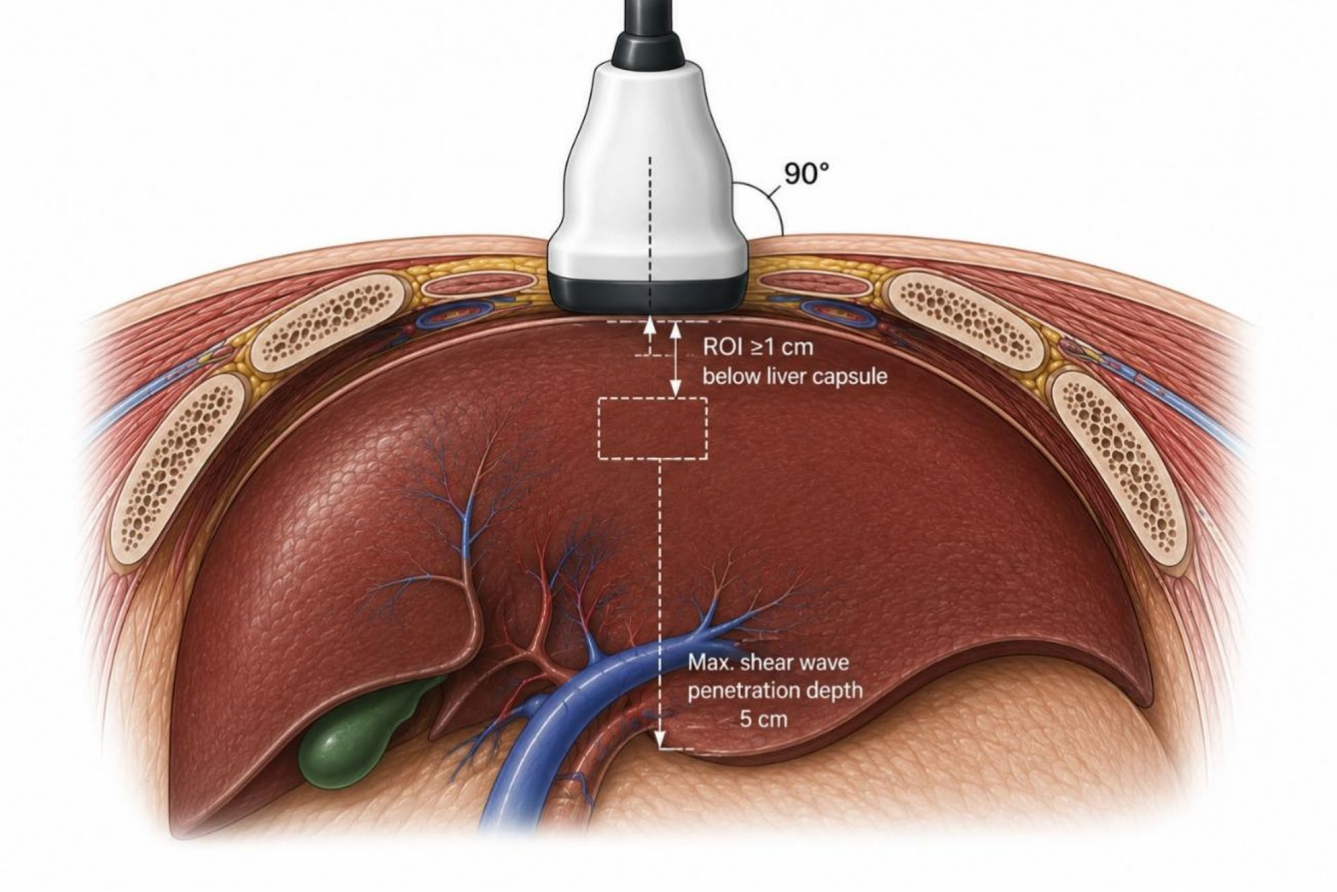
Schematic illustration of the pSWE measurement protocol. The ultrasound probe was positioned perpendicular to the liver capsule using an intercostal approach. The region of interest (ROI) was placed at least 1 cm below the liver capsule within the right liver lobe while avoiding large vessels and artifacts. The maximum penetration depth of the shear wave was 5 cm. Measurements were obtained during breath-hold in neutral respiration using acoustic radiation force impulse (ARFI)-based point shear wave elastography

A total of 100 consecutive patients fulfilled the study inclusion criteria and underwent liver biopsy and ultrasound examination. Of these, 59 patients had numerically evaluable Siemens pSWE measurements and complete histopathological assessment and therefore constituted the final study cohort for diagnostic accuracy analyses (Fig. 1).

The study was conducted in accordance with the Declaration of Helsinki and was approved by the Ethics Committee of the University of Ulm (approval no. 330/20) [13]. Written informed consent was obtained from all participants before study inclusion.

### Inclusion and exclusion criteria

Patients were classified according to the current MASLD nomenclature for the purpose of the present analysis. Patient recruitment and the clinical indication for liver biopsy followed the recommendations and clinical standards applicable at the time of enrolment [4]. Viral hepatitis was excluded by serological testing (HBs antigen, anti-HBc antibody, anti-HCV antibody; HAV and HEV serology if clinically indicated). Other chronic liver diseases, including autoimmune, cholestatic, genetic, drug-induced, and alcohol-related liver diseases, were excluded based on clinical history, laboratory testing, and diagnostic assessment. Alcohol consumption was assessed using the standardized AUDIT-C questionnaire [14]. Significant alcohol intake (≥ 140 g/week in women and ≥ 210 g/week in men) resulted in exclusion. Pregnant or lactating women and patients with insufficient biopsy quality were also excluded.

### Point shear wave elastography

Liver stiffness was assessed using point shear wave elastography (pSWE) based on acoustic radiation force impulse (ARFI) technology on a Siemens Acuson Sequoia ultrasound system. Examinations were performed using a standard convex transducer (1–6 MHz) without the use of specialized deep abdominal probes. Measurements were conducted under standardized conditions in accordance with international elastography guidelines [9, 10]. The measurement protocol is illustrated schematically in Fig. 1. Examiners were blinded to histological results and had extensive experience in abdominal ultrasound (> 10 years; >3000 examinations annually). Patients fasted for at least 6 h prior to examination. Measurements were performed in the supine position with the right arm in maximal abduction using an intercostal approach. The region of interest was placed in the right liver lobe, avoiding vessels, bile ducts, and focal lesions. Measurements were acquired during breath-hold in neutral respiration. In accordance with guideline recommendations, ten measurements were obtained whenever feasible. At least five valid measurements were required for inclusion in the analysis; in the majority of cases, ten valid measurements were available. The median shear wave velocity (m/s) was used for analysis. Values were additionally reported in kilopascal (kPa) for comparability with the literature.

### Histopathological assessment

Ultrasound-guided percutaneous liver biopsy was performed using a core needle technique. At least two biopsy cores were obtained per patient. Specimens were formalin-fixed, paraffin-embedded, and processed using standard histological techniques. Three experienced liver pathologists, blinded to elastography results, independently evaluated all samples. Discrepancies were resolved by consensus. Fibrosis staging (F0–F4) was performed according to the Brown and Kleiner (NASH CRN) classification and served as the histological reference standard for all diagnostic accuracy analyses. Histological features of steatosis, hepatocellular ballooning and lobular inflammation were additionally assessed according to the Bedossa SAF scoring system. Advanced fibrosis was defined as fibrosis stage ≥F3 (primary endpoint) [15, 16].

### Biopsy adequacy

Biopsy adequacy was confirmed before histopathological assessment by experienced liver pathologists. All included biopsy specimens had a cumulative tissue length of at least 10 mm. If fragmentation of the biopsy core occurred during tissue processing, the cumulative length of all tissue fragments was considered for adequacy assessment.

### Serum-based fibrosis markers

The Fibrosis-4 Index (FIB-4) was calculated using age, aspartate aminotransferase (AST), alanine aminotransferase (ALT), and platelet count [5].

### Study endpoints

The primary endpoint was the detection of advanced fibrosis (≥ F3) based on the Brown and Kleiner fibrosis stage. Secondary endpoints included the detection of significant fibrosis (≥ F2), the correlation between liver stiffness and histological fibrosis stage, the diagnostic accuracy of pSWE as assessed by AUROC, and the identification of clinically applicable rule-out and rule-in thresholds.

### Statistical analysis

The primary analysis evaluated the diagnostic performance of point shear wave elastography (pSWE) for the detection of advanced fibrosis (≥ F3), using histopathological fibrosis staging according to the Brown and Kleiner (NASH CRN) classification as the reference standard.

Diagnostic accuracy was assessed using receiver operating characteristic (ROC) analysis. The area under the ROC curve (AUROC) was calculated together with its corresponding 95% confidence interval (CI).

The Youden index was calculated as an exploratory measure to identify the threshold providing the best overall discrimination between patients with and without advanced fibrosis. In addition, clinically applicable rule-out and rule-in thresholds were predefined according to their intended clinical use rather than solely maximizing overall diagnostic accuracy. The rule-out threshold was selected to achieve a sensitivity of at least 95%, whereas the rule-in threshold was selected to achieve a specificity of at least 90%.

For both predefined thresholds, sensitivity, specificity, positive predictive value (PPV), and negative predictive value (NPV) together with their corresponding 95% confidence intervals were calculated.

Continuous variables are presented as median and interquartile range (IQR), whereas categorical variables are presented as absolute numbers and percentages. Comparisons between groups were performed using the Mann–Whitney U test for continuous variables and Fisher’s exact test or the χ² test for categorical variables, as appropriate.

The association between liver stiffness and histological fibrosis stage was evaluated using Spearman’s rank correlation coefficient.

Statistical analyses were performed using Python (NumPy, SciPy, and scikit-learn) [17]. All statistical tests were two-sided, and a p-value < 0.05 was considered statistically significant. Confidence intervals were calculated using exact binomial methods.

## Results

### Study population

A total of 59 patients with MASLD and available histological fibrosis staging and numerically evaluable pSWE measurements were included in the final analysis. Advanced fibrosis (≥ F3) was present in 18 of 59 patients (30.5%).

### Baseline characteristics

Baseline characteristics of the study population are summarized in Table 1. The median age of the overall cohort was 51.0 years (IQR 37.5–62.0), and 45.8% were female. Patients with advanced fibrosis were significantly older compared with those without advanced fibrosis. Platelet counts and albumin levels were significantly lower, whereas FIB-4 values and pSWE measurements were markedly higher in the ≥F3 group. Bilirubin levels were also significantly increased in patients with advanced fibrosis. In contrast, BMI, AST, ALT, and GGT did not show a clear discriminatory pattern between fibrosis groups.

**Table 1:** Baseline characteristics stratified by advanced fibrosis (≥ F3)

Characteristic	Total (*n* = 59)	< F3 (*n* = 41)	≥F3 (*n* = 18)	*p*-value*
Patients, n	59	41	18	
Sex, female	27 (45.8%)	20 (48.8%)	7 (38.9%)	0.576
Age, years	51.0 (37.5–62.0)	42.0 (36.0–57.0)	63.0 (52.8–71.5)	0.001
BMI, kg/m²	27.28 (24.20–31.77)	27.34 (23.96–32.55)	26.95 (24.70–29.21)	0.767
AST, U/L	55.0 (35.5–94.0)	48.0 (32.0–108.0)	56.0 (41.3–70.0)	0.908
ALT, U/L	61.5 (41.0–105.5)	67.0 (47.0–117.0)	55.0 (30.0–79.0)	0.112
GGT, U/L	151.0 (68.5–347.0)	151.0 (52.0–323.0)	161.0 (79.8–373.8)	0.474
Bilirubin, µmol/L	11.0 (7.0–16.0)	9.5 (6.0–13.3)	15.0 (11.0–20.0)	0.014
Albumin, g/L	44.0 (40.0–47.0)	46.0 (43.5–49.0)	40.0 (37.5–43.8)	< 0.001
Platelets, 10⁹/L	190.0 (129.0–258.0)	222.0 (164.0–283.0)	113.5 (89.8–173.0)	< 0.001
FIB-4	1.78 (0.92–4.17)	1.28 (0.88–2.35)	5.40 (2.84–7.18)	< 0.001
pSWE (median), m/s	1.26 (1.05–1.65)	1.13 (1.02–1.37)	2.21 (1.45–3.28)	< 0.001
pSWE (median), kPa	4.75 (3.30–8.23)	3.75 (3.10–5.60)	14.58 (6.25–32.40)	< 0.001

Data are presented as median (IQR) or n (%), unless otherwise indicated

*Continuous variables: Mann–Whitney U test; categorical variables: Fisher’s exact test

### Liver stiffness according to fibrosis stage

**Fig. 2:**
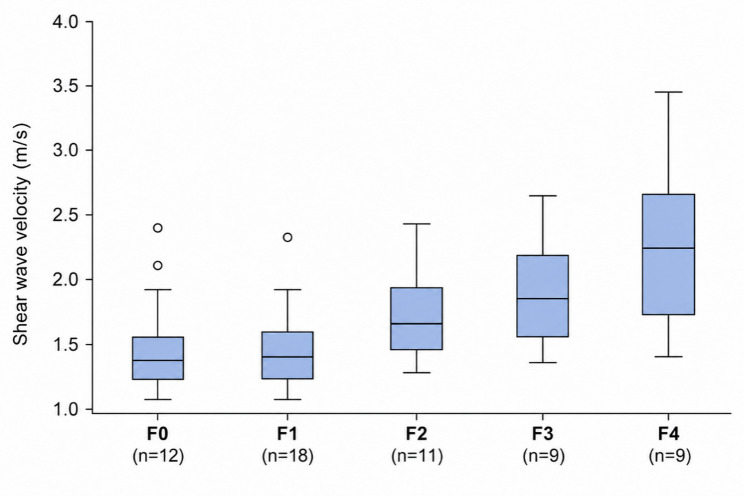
Distribution of pSWE values according to histological fibrosis stage. Distribution of pSWE values across histological fibrosis stages (Brown and Kleiner classification). Boxplots show the median, interquartile range, and range of liver stiffness measurements. Liver stiffness increased progressively with advancing fibrosis stage, with greater separation observed between non-advanced (F0–F2) and advanced fibrosis (F3–F4)

Median pSWE values increased progressively with advancing histological fibrosis stage. While patients with non-advanced fibrosis (F0–F2) showed overlapping liver stiffness values, a marked increase was observed in patients with advanced fibrosis (F3–F4), indicating a clear separation between clinically relevant fibrosis stages. Shear wave velocity correlated significantly with histological fibrosis stage (Spearman ρ = 0.68, *p* < 0.001). The distribution of pSWE values across fibrosis stages is illustrated in Fig. 2. The distribution of histopathological fibrosis stages according to the Brown and Kleiner classification is summarized in Table 2.

**Table 2:** Histopathological reference standard of the final study cohort (*n* = 59)

Histopathological characteristic	*n* (%)
Patients with advanced fibrosis (≥ F3)	18 (30.5%)
Fibrosis stage (Brown & Kleiner)	
F0	12 (20.3%)
F1	18 (30.5%)
F2	11 (18.6%)
F3	9 (15.3%)
F4	9 (15.3%)

Fibrosis staging was performed according to the Brown and Kleiner (NASH Clinical Research Network) classification. Data are presented as absolute numbers and percentages

### Diagnostic accuracy for advanced fibrosis

**Fig. 3:**
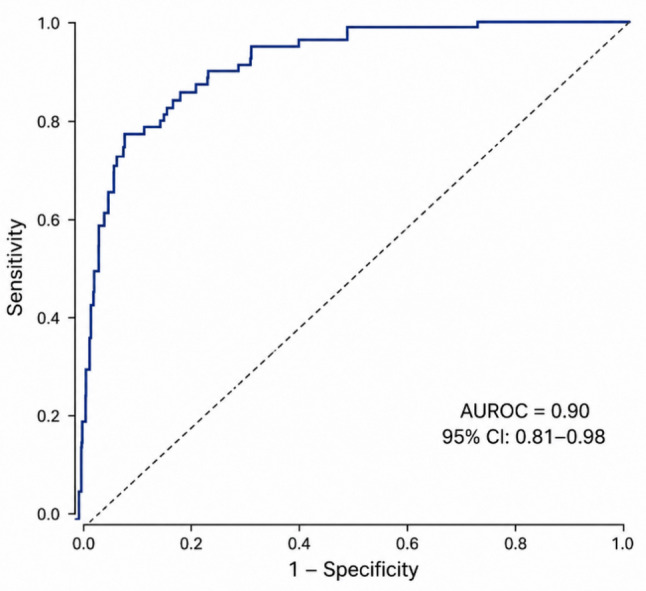
Receiver operating characteristic (ROC) curve for the detection of advanced fibrosis (≥ F3). Receiver operating characteristic (ROC) analysis of point shear wave elastography (pSWE) for the detection of advanced fibrosis (≥F3), demonstrating excellent diagnostic performance (AUROC 0.90; 95% CI 0.82–0.97)

Receiver operating characteristic (ROC) analysis demonstrated excellent diagnostic performance of pSWE for the detection of advanced fibrosis (≥ F3), with an AUROC of 0.90 (95% CI 0.82–0.97). The ROC curve for pSWE is shown in Fig. 3.

### Clinically applicable rule-in and rule-out thresholds

**Fig. 4:**
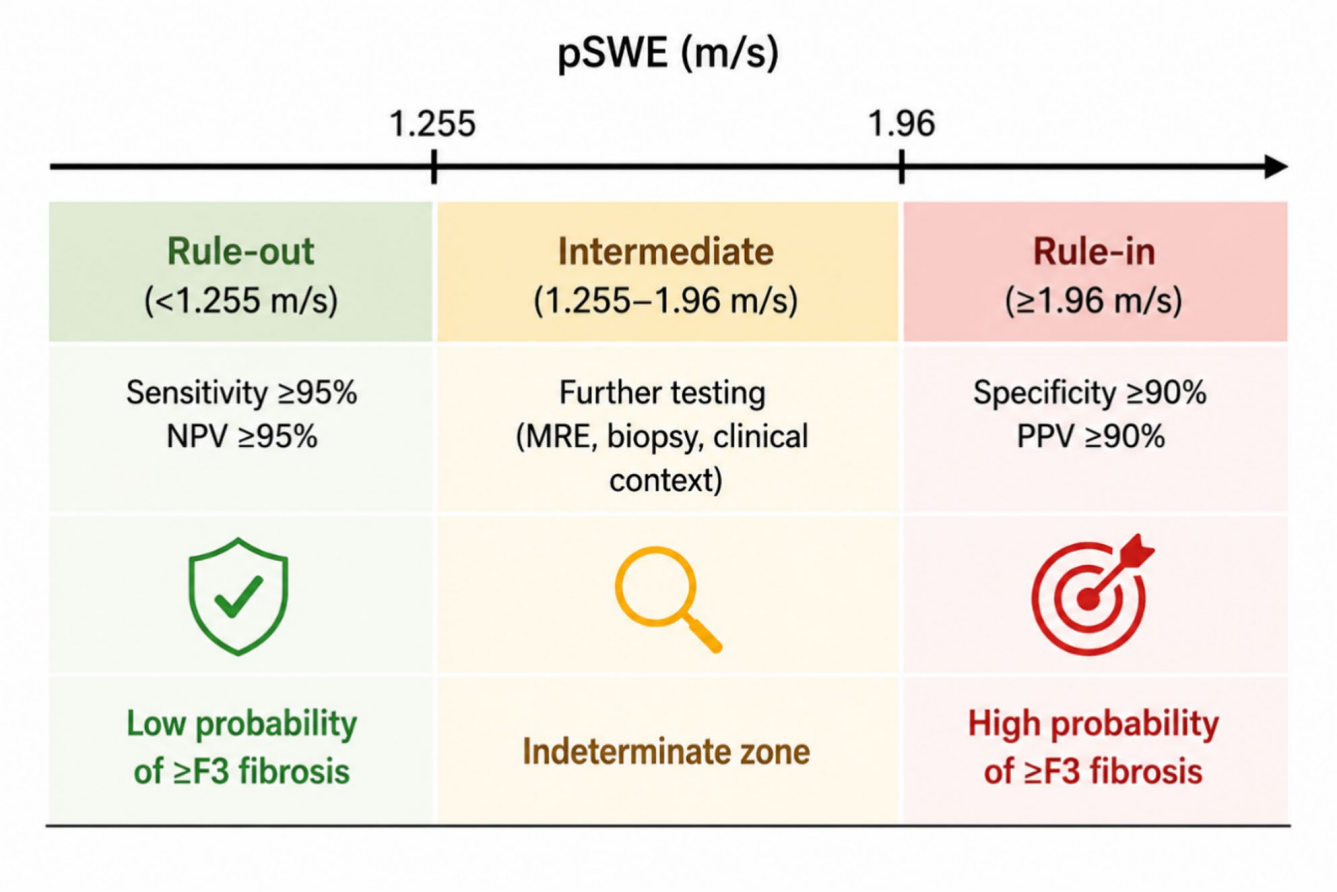
Clinically applicable pSWE thresholds for the detection of advanced fibrosis (≥ F3). Values below 1.255 m/s define the rule-out zone, whereas values of 1.96 m/s or higher define the rule-in zone. Measurements between both thresholds represent an intermediate diagnostic zone in which additional clinical, laboratory, or non-invasive assessment should be considered

A threshold of 1.255 m/s defined a rule-out zone for advanced fibrosis, achieving 100% sensitivity and a 100% negative predictive value. Conversely, a threshold of 1.96 m/s defined a rule-in zone, yielding 97.6% specificity and a 91.7% positive predictive value. Diagnostic performance estimates for both predefined thresholds, including their corresponding 95% confidence intervals, are summarized in Table 3. Measurements between these thresholds represented an intermediate diagnostic zone requiring further clinical, laboratory, and non-invasive assessment. A graphical representation of the proposed clinical decision framework is shown in Fig. 4.

**Table 3:** Diagnostic performance of clinically applicable pSWE rule-out and rule-in thresholds for advanced fibrosis (≥ F3)

Threshold (m/s)	Sensitivity % (95% CI)	Specificity % (95% CI)	PPV % (95% CI)	NPV % (95% CI)	Clinical interpretation
1.255 (Rule-out)	100.0 (81.5–100.0)	68.3 (51.9–81.9)	58.1 (39.1–75.5)	100.0 (87.7–100.0)	Advanced fibrosis can be reliably excluded.
1.96 (Rule-in)	61.1 (35.7–82.7)	97.6 (87.1–99.9)	91.7 (61.5–99.8)	85.1 (71.7–93.8)	Advanced fibrosis is highly likely.

Diagnostic thresholds were predefined according to their intended clinical application rather than optimized using the Youden index. The rule-out threshold was selected to achieve a sensitivity of at least 95%, whereas the rule-in threshold was selected to achieve a specificity of at least 90%

*PPV* Positive predictive value, *NPV* Negative predictive value, *CI* Confidence interval

## Discussion

In this prospective, histologically validated MASLD cohort, point shear wave elastography (pSWE) demonstrated excellent diagnostic performance for the detection of advanced fibrosis, with an AUROC of 0.90. Importantly, the identification of distinct rule-in and rule-out thresholds enables a clinically intuitive interpretation of pSWE measurements, as illustrated in Fig. 4. This approach facilitates direct translation into clinical decision-making by distinguishing patients with a low probability of advanced fibrosis from those with a high likelihood, while acknowledging an intermediate diagnostic zone. These findings are consistent with previous studies demonstrating the diagnostic utility of pSWE in MASLD populations. In particular, recent data from larger cohorts have reported high diagnostic accuracy of pSWE for advanced fibrosis. In a retrospective study by Sun et al., multisample pSWE achieved an AUROC of 0.96 in obese patients with MASLD. However, important methodological differences should be considered. While the study by Sun et al. was retrospective in design, our study was conducted prospectively with standardized data acquisition, reducing the risk of selection and measurement bias [12]. Further support for the clinical applicability of pSWE is provided by recent work from Dominik et al., demonstrating the value of ultrasound-based multiparametric assessment in obese MASLD patients [11]. In contrast to imaging-focused approaches, our study additionally incorporated the FIB-4 index, enabling a clinically relevant comparison between serum-based and imaging-based non-invasive tests in line with current guideline-recommended stepwise diagnostic algorithms. A key strength of the present study is the use of histology as the reference standard with independent evaluation by three experienced liver pathologists. Consensus-based histological assessment reduces interobserver variability and strengthens the robustness of fibrosis staging. Together with the prospective study design, this enhances the overall reliability and clinical relevance of our findings.

From a clinical perspective, pSWE offers important advantages due to its integration into routine ultrasound systems, allowing broad availability and easy implementation. The defined rule-out threshold enables reliable exclusion of advanced fibrosis, while the rule-in threshold supports identification of high-risk patients (Table 3). The intermediate zone reflects the need for combined interpretation with clinical and laboratory parameters, consistent with current guideline recommendations.

The proposed dual-threshold approach should be interpreted as a clinically applicable decision aid rather than a set of absolute diagnostic cut-offs. Liver stiffness measurements obtained by pSWE are subject to biological and technical variability, as described in current elastography guidelines. The separation between the proposed rule-out and rule-in thresholds should therefore be interpreted as a pragmatic clinical decision framework rather than as absolute diagnostic boundaries for individual measurements. Furthermore, the present study included a moderate number of patients with advanced fibrosis, resulting in uncertainty around the estimated diagnostic performance measures despite the excellent observed point estimates. Therefore, the proposed thresholds should be interpreted within the overall clinical context and require external validation in larger independent cohorts before widespread implementation.

An important technical aspect relates to the measurement approach. In contrast to some recent studies using specialized deep abdominal transducers (DAX) to improve feasibility in obese patients, measurements in our study were performed using a standard convex probe. While DAX transducers may increase measurement success rates in challenging patient populations, potential differences in measurement characteristics should be considered, and direct transferability of thresholds may be limited.

Several limitations should be acknowledged. First, this was a single-center study, which may limit generalizability. Second, although histology was used as the reference standard, liver biopsy is subject to sampling variability. Third, the sample size was moderate, although comparable to other histologically validated elastography studies. In addition, intra- and interobserver repeatability of pSWE measurements was not assessed in the present study and therefore cannot be evaluated from our data.

## Conclusion

Point shear wave elastography provides reliable and clinically applicable non-invasive detection of advanced fibrosis in MASLD. Technique-specific thresholds enable meaningful rule-in and rule-out strategies and support integration of pSWE into stepwise non-invasive diagnostic pathways.

## Data Availability

All data supporting the findings of this study are available within the paper.
